# Expression of GPR43 in Brown Adipogenesis Is Enhanced by Rosiglitazone and Controlled by PPAR*γ*/RXR Heterodimerization

**DOI:** 10.1155/2018/1051074

**Published:** 2018-05-16

**Authors:** Jiamiao Hu, Arong Zhou, Peter C. K. Cheung, Baodong Zheng, Shaoxiao Zeng, Shaoling Lin

**Affiliations:** ^1^College of Food Science, Fujian Agriculture and Forestry University, Fuzhou, Fujian 350002, China; ^2^Warwick Medical School, University of Warwick, Coventry, West Midlands, UK; ^3^School of Life Sciences, The Chinese University of Hong Kong, Shatin, New Territories, Hong Kong

## Abstract

GPR43, a G-protein coupled receptor recognizing short-chain fatty acids, has been reported to participate in many biological functions of white adipocytes, such as adipogenesis and lipolysis. However, the functional role of GPR43 in brown adipocytes is still not clear. In this study, we investigated the effects of the PPAR*γ* agonist rosiglitazone on GPR43 expression in brown adipogenesis. The results demonstrated that GPR43 was expressed during the late phase of brown adipocyte differentiation, which could be further augmented by adipogenic agent rosiglitazone treatment. The PPAR*γ*/RXR heterodimerization was found to be the key transcription factor for this enhancing effect of rosiglitazone on GPR43 expression. Taken together, these results suggested GPR43 levels might be regulated by PPAR*γ*-activated events during brown adipocytes differentiation and reflect the adipogenesis status of brown adipocytes.

## 1. Introduction

In the last decades, several G-protein coupled receptors (GPCRs), i.e., GPR40, GPR41, GPR43, GPR84, and GPR120, were deorphaned as free fatty acids (FFAs) receptors [1–5]. Of these receptors, GPR43 is activated by short-chain fatty acids such as acetate, propionate, and butyrate [2–4]. RT-PCR analyses have demonstrated that GPR43 is expressed abundantly in spleen, bone marrow, liver, intestine, and adipose tissue [4, 6, 7].

So far, several independent studies have indicated a potential link between GPR43 expression and white adipogenesis. For example, GPR43 levels were significantly increased during the differentiation of 3T3-L1 adipocytes, which can be further upregulated by treatment with adipogenic agent such as troglitazone [8]. In mice fed with a high-fat diet (HFD), the augmented adiposity and adipocyte enlargement were observed with an overexpression of GPR43 in the subcutaneous white adipose tissue [6, 9]. In contrast, administration of inulin-type fructans could counteract this HFD-induced GPR43 overexpression and peroxisome proliferator activated receptor *γ*- (PPAR*γ*-) related adipogenesis in the white adipose tissue of mice [9], suggesting GPR43 expression levels in white adipose tissue might reflect the obesity status in the animal's body. Furthermore, functional analyses have also suggested the promotive effects of short-chain fatty acids on adipogenesis* via* GPR43 in white adipocytes [8, 10]. However, up to date, most studies mainly focus on the potential functions of GPR43 in white adipocytes and the importance of GPR43 expression in brown adipocytes has not yet been thoroughly studied.

Recently, the promoter regions of GPR43 were determined in human monocytes, showing transcription factors including XBP1 were key transcription factors for the regulation of GPR43 expression [11]. Notably, transcription factor XBP1 was also found to play crucial roles in the adipogenesis [12–15]. In addition, the proadipogenic effects of short-chain fatty acids in brown adipogenesis were also reported by independent studies both* in vitro* and* in vivo *[16, 17]. These evidences together suggest the possible link between GPR43 expression with brown adipogenesis and its expression levels might also reflect the status of adipogenesis.

To test this hypothesis, in the present study we investigated the expression pattern of GPR43 during the differentiation of brown adipocyte precursor cells. We also examined the effect of adipogenic agent rosiglitazone on the regulation of GPR43 transcription in brown adipocytes to understand more about the possible underlying mechanism.

## 2. Methods and Materials

### 2.1. Materials

Rosiglitazone was purchased from Sigma-Aldrich; PPAR*γ* antagonist GW9662 and RXR antagonist HX531 were purchased from Tocris Bioscience.

### 2.2. Cell Culture

The immortalized brown adipocyte cell line (IM-BAT) was constructed and a gift by Dr. Mark Christian (University of Warwick). Briefly, the primary brown preadipocytes were isolated from mice interscapular brown adipose tissue and cultured in DMEM/F12 medium containing 10% fetal bovine serum (FBS) and 1% antibiotic-antimycotic for 2 days before being immortalized by retroviral-mediated expression of temperature-sensitive SV40 large T antigen H-2kb-tsA58. Cells were continually cultured at 33°C and selected with G418 (100 mg/ml) for 2 weeks and maintained in 50 *μ*g/ml of G418. IM-BAT cells were differentiated according to previous report [16] by treating with 500 *μ*M 3-isobutyl-1-methylxanthine (IBMX), 250 nM dexamethasone, 170 nM insulin, and 1 nM 3,3′,5-triiodo-l-thyronine (T3) in DMEM/F12 medium containing 10% FBS for 2 days, followed by incubation with DMEM/F12 medium containing 170 nM insulin, 1 nM T3, and 10% FBS till clear lipid droplets could be seen under microscopy. Rosiglitazone, GW9662, or HX531 was dissolved in DMSO and then directly diluted in DMEM/F12 and treated during the course of differentiation. The final concentration of the DMSO did not exceed 0.1% for either the control or the treated cells for all experiments. All experiments were performed below passages 22.

The white adipocytes 3T3-L1 cells were purchased from ATCC and cultured in DMEM medium containing 10% newborn calf serum (NBCS). Cells were differentiated by treating with DMEM medium containing 500 *μ*M IBMX, 1 *μ*M dexamethasone, 1 *μ*g/ml insulin, and 10% FBS for 2 days, followed by incubation with DMEM medium containing 1 *μ*g/ml insulin and 10% FBS till lipid droplets were formed.

### 2.3. siRNA Transfection

To introduce siRNA into undifferentiated IM-BAT cells, siRNA was transfected with lipofectamine RNAiMAX according to manufacturer's manual. Briefly, IM-BAT cells were seeded to be 60–80% confluent at the time of transfection in DMEM:F12 medium containing 10% FBS without antibiotics. siRNA-lipid complexes were prepared and incubated at room temperature for 10 min before being added to cells. The cell culture medium was changed back into DMEM:F12 containing 10% FBS and 1% penicillin/streptomycin after 6 h. 24 h after transfection, the cells were differentiated as described above. The efficiency of gene knockdown was measured by quantitative real-time PCR.

### 2.4. Oil Red O Staining

Lipid accumulation of differentiated adipocytes was visualized and determined by Oil Red O staining kit (ECM950, Millipore). Briefly, differentiated adipocytes were washed with PBS and fixed in 3.7% formaldehyde for 15 minutes, followed by staining with Oil Red O solution (3 g/L) for 15 minutes. After staining, cells were washed twice with water and photographed using a microscope (Axiovert 40CFL, Olympus).

### 2.5. Quantitative Real-Time PCR

Total RNA was extracted by using GenElute Mammalian Total RNA Miniprep Kit (Sigma). Complementary DNAs were synthesized by reverse transcription of 1 *μ*g total RNA as templates using nanoScript 2 Reverse Transcription kits (Primerdesign). qRT-PCR was performed with Abi 7500 Fast Real-Time PCR System using SensiFAST SYBR (BIOLINE). Specific primers were designed in an intron-spanning manner for all possible cases. To avoid gDNA contamination during RNA extraction process, all RNA samples were treated with Precision DNase kits (Primerdesign). All primer pairs were confirmed not to self-dimerize by qRT-PCR using a nontemplate control. Expression levels were calculated according to the 2^−ΔΔCt^ method. The identity and purity of the amplified product were checked by analysing the melting curves carried out at the end of amplification.

### 2.6. Statistical Analysis

Results are presented as the mean ± SEM of at least triplicate samples in each experimental group; experiments were replicated to ensure consistency. Statistical significance of difference was determined using Student's *t*-test when comparing 2 groups or one-way ANOVA followed by post hoc Tukey's multiple comparison test when comparing more than 3 groups. Values were considered to be statistically significant if their *P* value was less than 0.05. All statistical calculations were analysed in GraphPad Prism 5.

## 3. Results

### 3.1. Rosiglitazone Treatment Significantly Increases GPR43 Expression in Brown Adipogenesis

**Figure 1:**
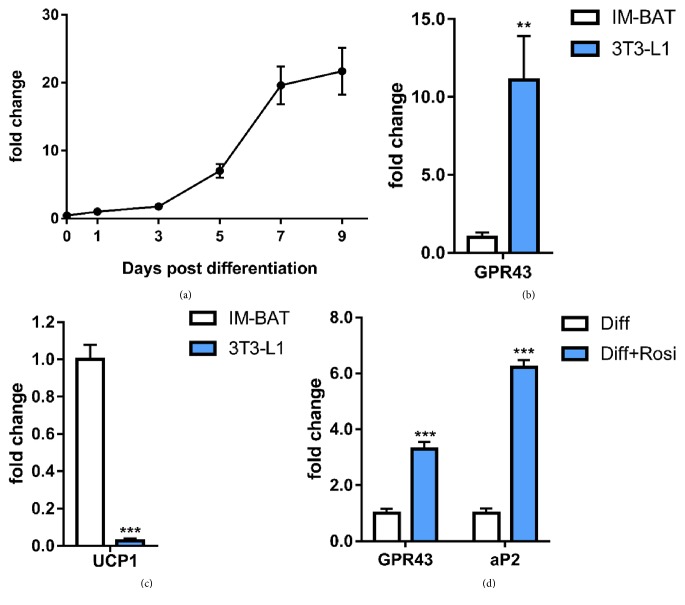
*Rosiglitazone treatment induces the increase of GPR43 transcription during the adipogenesis of brown adipocytes*. (a) mRNA transcription levels of GPR43 during the differentiation of IM-BAT adipocytes measured by quantitative real-time PCR. (b and c) Comparison of GPR43 (b) and UCP-1 (c) mRNA levels in mature IM-BAT adipocytes and 3T3-L1 adipocytes after 7 days of differentiation. (d) IM-BAT cells were differentiated with or without rosiglitazone (1 *μ*M) from day 3 to day 7 before GPR43 and aP2 mRNA levels were measured on day 7 after differentiation. Data are expressed as mean ± SEM (*n* = 3). ^*∗∗*^*P* < 0.01 and ^*∗∗∗*^*P* < 0.001 by Student's *t*-test.

We first measured the expression of GPR43 during the course of brown adipocytes differentiation by quantitative real-time PCR. Consistent with previous reports [16, 18], GPR43 mRNA expression was scarcely detected in the undifferentiated cells at day 0 but increased continuously throughout the differentiation period and was maintained at a high level after day 5 after differentiation (Figure 1(a)). Besides, our results also indicated that GPR43 expression in the differentiated brown adipocytes was much less (around 10-fold lower) than that in mature 3T3-L1 cells (white adipocytes cell line) while the levels of UCP1 were significantly higher in immortalized brown adipocytes (Figures 1(b) and 1(c)), showing that the brown adipocyte precursor cells are successfully differentiated.

To further investigate the relationship between brown adipogenesis and GPR43 expression, the effects of the adipogenic agent rosiglitazone on GPR43 expression in brown adipocytes were evaluated. The results demonstrated that rosiglitazone treatment during differentiation induced a significant increase (~3.3-fold) in GPR43 expression compared to the normal differentiation condition in mature brown adipocyte (Figure 1(d)). Adipocyte Protein 2 (aP2), a PPAR*γ* target gene known as a marker of adipocyte differentiation, also had ~6.2-fold increase in mature brown adipocytes treated with rosiglitazone, confirming the expected strong PPAR*γ* activation induced by rosiglitazone (Figure 1(d)).

### 3.2. Rosiglitazone Upregulating GPR43 Expression Requires PPAR*γ* and RXR Dimerization

**Figure 2:**
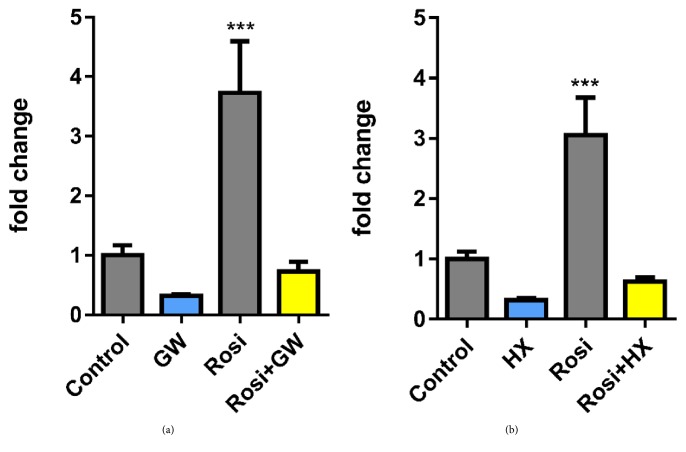
*Rosiglitazone-induced increase of GPR43 transcription requires dimerization of PPAR and RXR in brown adipocytes*. Undifferentiated IM-BAT cells were differentiated in IBMX, dexamethasone, insulin, and T3 containing DMEM:F12 medium for 2 days, followed by insulin and T3 containing medium in the absence or in the presence of rosiglitazone (1 *μ*M) as indicated. The PPAR*γ* antagonist GW9662 (1 *μ*M) (a) or RXR antagonist HX531 (1 *μ*M) (b) were also added to insulin and T3 containing medium during the course of differentiation from day 3 to day 7 after differentiation. mRNA transcription levels of GPR43 in mature adipocytes were measured by quantitative real-time PCR. Data are expressed as mean ± SEM (*n* = 3). ^*∗∗∗*^*P* < 0.01 by one-way ANOVA followed by Tukey's multiple comparison.

Studies have identified that the effects of rosiglitazone in adipocytes could be divided into PPAR*γ*-dependent and PPAR*γ*-independent ones. To elucidate the involvement of PPAR*γ* in rosiglitazone-induced increase in GPR43 expression, PPAR*γ* selective antagonist GW9662 was cotreated with rosiglitazone during the differentiation of brown adipocytes. The results indicated significant upregulation of GPR43 expression was only observed in cells treated with rosiglitazone but not in cells cotreated with GW9662, suggesting the upregulated GPR43 expression by rosiglitazone is PPAR*γ* dependent (Figure 2(a)).

Similarly, RXR homo- and heterodimer antagonist HX531 was also applied to test the role of heterodimerization of PPAR*γ* and RXR in GPR43 expression. The results showed that cotreatment of HX531 with rosiglitazone also effectively inhibited rosiglitazone-induced GPR43 expression in brown adipocytes (Figure 2(b)). These evidences together suggested that the increase of GPR43 expression induced by rosiglitazone may require the formation of heterodimerization between PPAR*γ* and RXR.

### 3.3. Rosiglitazone Overcomes the Effects of XBP1 Knockdown on GPR43 mRNA Expression in Brown Adipocyte

XBP1 has been recently identified as the key transcription factor for the expression of GPR43 in human monocytes [11]. Therefore, we next identified the role of XBP1 in rosiglitazone-induced increase of GPR43 expression in brown adipocytes. We firstly knocked down the expression of XBP1 in brown preadipocytes and then differentiated the knocked-down cells with or without rosiglitazone till day 5 as described above. To make sure of the duration of silencing after siRNA transfection, the expression of XBP1 was checked from day 1 to day 5 after differentiation. The XBP1 mRNA transcription levels were checked by real-time PCR and compared to cells transfected with control siRNA. The result showed that the knockdown efficiency reached >60% at 48 h after transfection (24 h after differentiation). Moreover, the knockdown efficiency kept at >70% until day 5 after differentiation (Supplementary Figure 1), indicating the XBP1 gene silencing effect can last through the differentiation process of IM-BAT cells.

**Figure 3:**
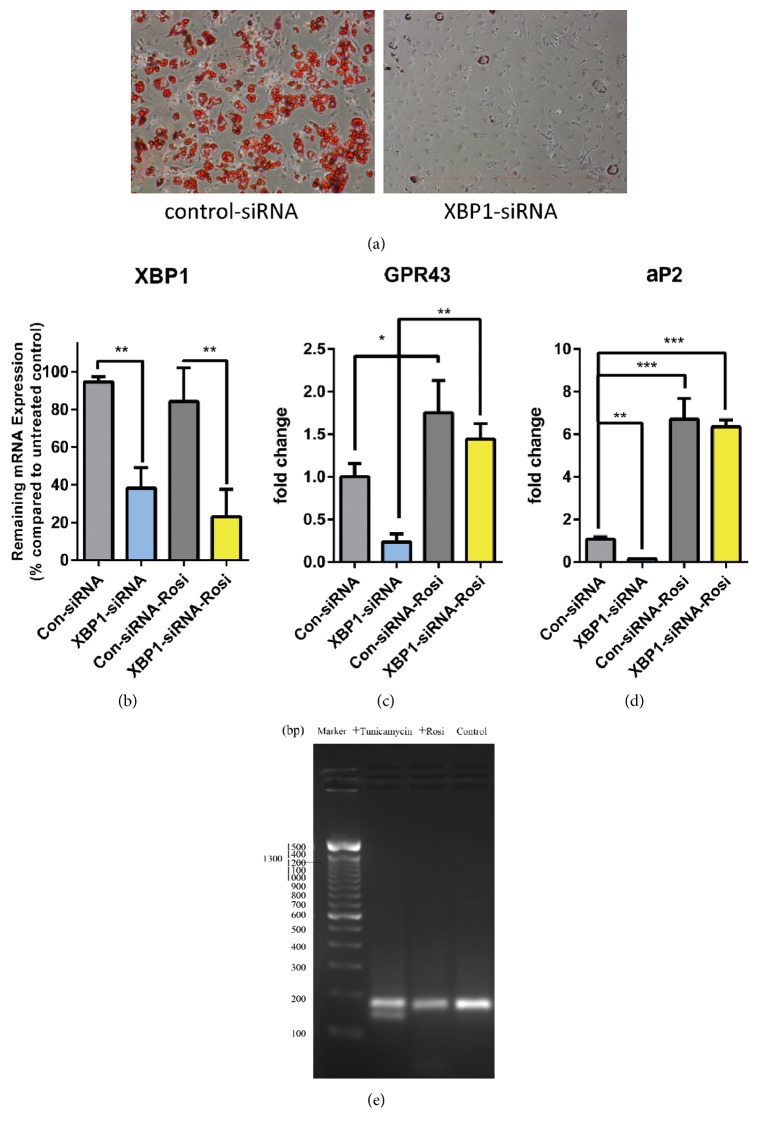
*Rosiglitazone-mediated increase of GPR43 transcription in adipocytes is XBP1 independent*. (a–d) IM-BAT adipocytes transfected with control siRNA or XBP1 siRNA were differentiated in the absence or in the presence of rosiglitazone (1 *μ*M) during differentiation from day 3 to day 5 after differentiation. Accumulated lipids in the siRNA transfected IM-BAT adipocytes were measured by Oil Red O staining (a), while mRNA levels of XBP1 (b), GPR43 (c), and aP2 (d) in the transfected IM-BAT adipocytes were measured by quantitative real-time PCR. Data are expressed as mean ± SEM (*n* = 3). ^*∗*^*P* < 0.05; ^*∗∗*^*P* < 0.01;  ^*∗∗∗*^*P* < 0.001 by one-way ANOVA followed by Student's *t*-test. (e) Agarose gel of undifferentiated IM-BAT adipocytes treated with rosiglitazone (1 *μ*M) for 6 h. Splicing of XBP1 mRNA was detected by RT-PCR. cDNA from preadipocytes treated with tunicamycin was used as positive control.

In accordance with previous findings, knockdown of XBP1 (Figure 3(b)) significantly impairs the differentiation of preadipocytes as demonstrated by oil accumulation (Figure 3(a)) as well as adipocyte differentiation marker aP2 transcription (Figure 3(d)). Accordingly, the expression level of GPR43 also significantly decreased in XBP1-knockdown cells (Figure 3(c)). Interestingly, in brown adipocytes transfected siRNA targeting XBP1, the rosiglitazone still led to a significant increase in GPR43 expression, indicating rosiglitazone can overcome the effects of the loss of XBP1 on GPR43 transcription (Figure 3(c)). Besides, we also tested the effect of rosiglitazone treatment on XBP1 splicing. The results also showed that there was no obvious XBP1 mRNA splicing detected (Figure 3(e)), suggesting rosiglitazone treatment has little effects on XBP1 activation. Taken together, these results suggest that rosiglitazone-induced GPR43 expression in brown adipocytes might be mediated by XBP1-independent mechanism.

## 4. Discussion

It has been demonstrated that the activation of GPR43 in white adipose tissue promotes adipocyte differentiation and drives the inhibition of lipolysis [19]. However, little information has been known about the importance of this receptor in brown adipose tissue. Here, our results indicated that GPR43 expression was initiated in brown adipocytes from day 3 of postinduction period, while in white adipocytes abundant GPR43 expression was also observed in the late phase of differentiation [8]. Moreover, our results also demonstrated that the GPR43 expression was rapidly and consistently increased by rosiglitazone treatment in brown adipocytes, which is also similar to the effects of troglitazone in white adipocytes reported in previous studies [8]; these findings support the hypothesis that GPR43 expression in brown adipocytes might share a similar mechanism to that in white adipocytes during adipogenesis.

Rosiglitazone, a well-known antidiabetic drug, can upregulate the activities of PPAR-*γ* in many peripheral tissues (including adipose tissue) [20]. Since PPAR*γ* is most highly expressed in adipose tissue and plays a crucial role as an adipogenic regulator during adipogenesis, rosiglitazone was also found to be a highly active adipogenic agent, which promotes adipocyte differentiation and activates adipocyte-specific genes expression [20]. Here, we observed that the GPR43 expression was significantly upregulated by rosiglitazone in brown adipocytes. Furthermore, our results also showed that the disruption of PPAR*γ* and RXR heterodimerization almost abolished the rosiglitazone-induced increase in GPR43 expression in brown adipocytes, indicating the positive role of PPAR*γ* activation in GPR43 expression in brown adipocytes.

XBP1 has been elucidated as a core* cis* element controlling the GPR43 transcription in human monocytes [11]. Here, our results also demonstrated that knockdown of XBP1 significantly impaired the expression of GPR43 in brown adipocytes. However, it seemed that rosiglitazone-induced augment of GPR43 expression was not significantly affected by the knockdown of XBP1. Indeed, previous evidence also suggested that XBP1 does not seem to be necessary for the proadipogenic effect of thiazolidinedione. For example, although deletion of XBP1 inhibited adipogenesis in adipocytes* in vitro*, such inhibitory effect could be overcome by thiazolidinediones [21]. Moreover, deletion of adipocyte-XBP1* in vivo* did not affect body weight, adipose tissue mass, serum insulin, or glucose homeostasis, indicating XBP1 is not a contributing factor to the formation or expansion of adipose tissue* in vivo *[21]. Here, our findings are consistent with these previous results in that the increase of GPR43 expression by rosiglitazone seemed to be mediated at least partially by XBP1-independent mechanism.

In addition, the fact that short-chain fatty acids mediate a wide range of metabolic functions in white adipocytes and white adipose tissues* via* the GPR43 receptors [8, 19, 22] strongly supports the hypothesis that GPR43 acts as sensor that regulates energy metabolism in white adipose tissue. Brown adipose tissue also can regulate the metabolism and energy homeostasis by heat production when the body is exposed to cold temperature [23, 24]. Indeed, brown adipose tissue plays an important role in maintaining body temperature in rodentine mammals and infants. Recent studies also confirmed the presence of brown adipose tissue in human adults [25]. This discovery has drawn great interest in investigating its potential therapeutic application. Indeed, studies already suggested the regulation of GPR43 in brown adipocytes. It has been found that butyrate, an agonist of GPR43, increased* in vivo* adaptive thermogenesis, mitochondrial biogenesis, and UCP-1 expression in brown adipose tissue in mice [26], while HFD-fed GPR43 knock-out mice had significant lipid droplets in intrascapular brown adipose tissue compared to HFD-fed WT mice [27]. Recently, GPR43 agonist acetate and propionate were also found to prevent diet-induced metabolic disorders by induction of Cidea and mitochondrial marker Tfam expression as well as increasing Nrg4 secretion in brown adipose tissue, respectively [28], highlighting the potential links between the activation of GPR43 by short-chain fatty acids in brown adipose tissue and the regulatory effects on metabolism.

Notably, a previous study has reported that chronic PPAR*γ* stimulation led to thermogenic gene expression in a subset of white precursor cells (Hoxc9 positive but Myf5 negative) from epididymal white adipose tissue depot [29]. Meanwhile, GPR43 activation has also been demonstrated to promote the beige adipogenesis* in vivo *[30]. Since PPAR*γ* activation also contributes to the increase in GPR43 expression during brown adipogenesis, these evidences together imply a hypothesis that GPR43 expression driven by PPAR*γ* activation might further enhance the effects of PPAR*γ* stimulation on beige adipogenesis. However, this hypothesis needs further studies to be proven.

In summary, our results have shown that the expression of the short-chain fatty acids sensing GPR43 is initiated during the late phase of brown adipocyte differentiation and its expression levels can be used to reflect the status of brown adipocytes differentiation. GPR43 expression can be increased by the treatment of adipogenic agent such as rosiglitazone and controlled by PPAR*γ*/RXR heterodimerization. Our findings that the levels of GPR43 expression were associated with the differentiation stages of brown adipocyte may provide some important insights into the complex roles of GPR43 in the brown adipogenesis. These results also suggest the functional roles of GPR43 in regulating metabolism in mature adipocytes.

## Data Availability

The data used to support the findings of this study are available from the corresponding author upon request.

## Supplementary Materials

Supplementary Material Supplementary Figure 1: duration of XBP1 silencing after XBP1 siRNA transfection. IM-BAT cells were transfected with XBP1 siRNAs (Santa Cruz, no. sc38628) or control siRNA (sc-37007) at 200 nM. On the next day, cells were differentiated as indicated. Cells were lysed on day 0 through day 5 after differentiation and XBP1 transcription levels were measured using the real-time PCR. Knockdown efficiency is expressed relative to expression from cells transfected with control siRNA.
