# Supplementary material for: Expression of GPR43 in Brown Adipogenesis Is Enhanced by Rosiglitazone and Controlled by PPARγ/RXR Heterodimerization
Source: PPAR Res. 2018 May 16;2018:1051074. doi: 10.1155/2018/1051074 (PMC5976906; doi:10.1155/2018/1051074)

**Supplementary figure 1. Duration of XBP1 silencing after XBP1 siRNA transfection.** IM-BAT cells were transfected with XBP1 siRNAs (Santa Cruz, # sc38628) or Control siRNA (sc-37007) at 200 nM. The next day, cells were differentiated as indicated. Cells were lysed day 0 through day 5 post-differentiation and XBP1 transcription levels were measured using the real-time PCR. Knock-down efficiency is expressed relative to expression from cells transfected with control siRNA.


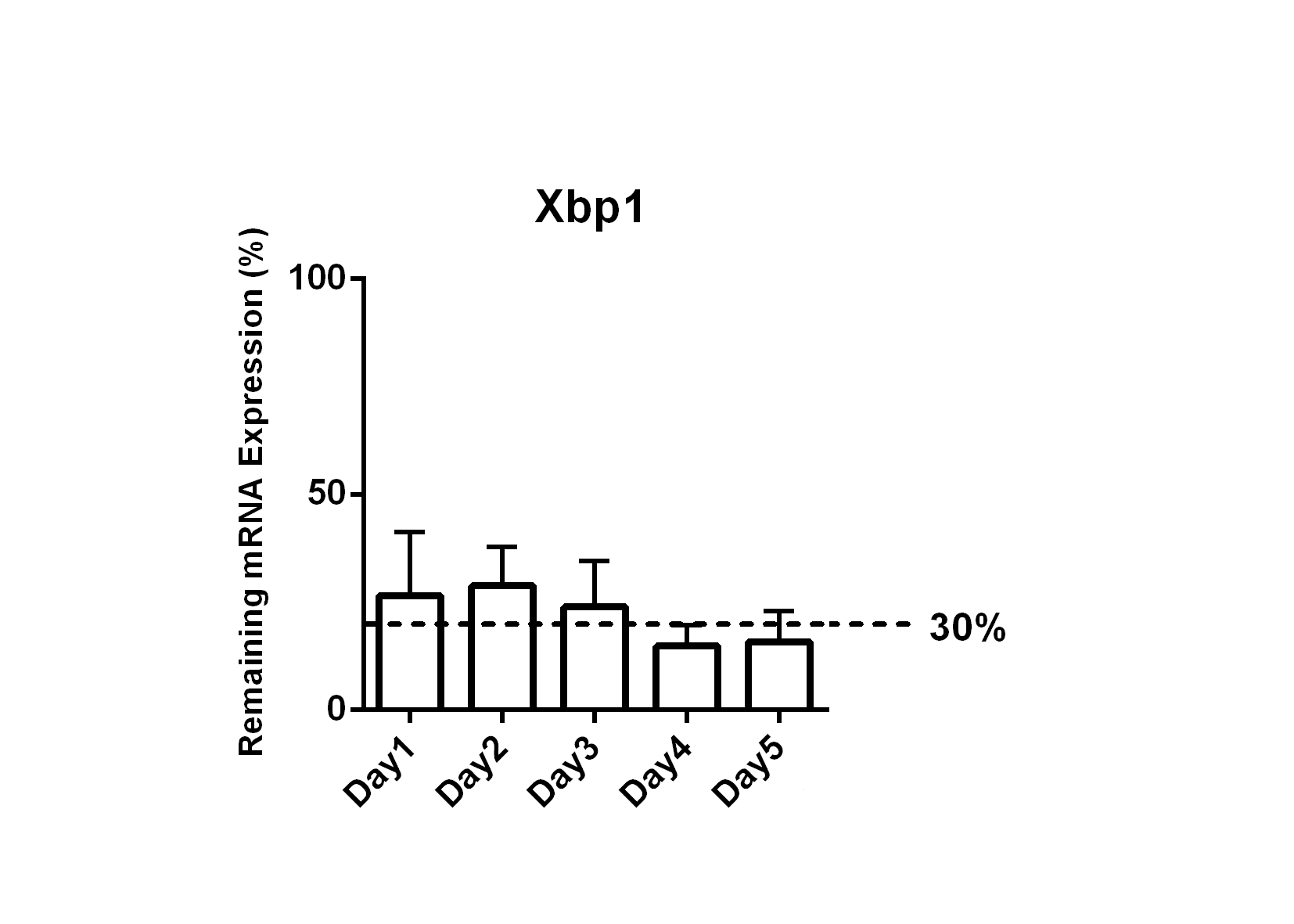

Supplement: Supplementary Material — Supplementary Figure 1: duration of XBP1 silencing after XBP1 siRNA transfection. IM-BAT cells were transfected with XBP1 siRNAs (Santa Cruz, no. sc38628) or control siRNA (sc-37007) at 200 nM. On the next day, cells were differentiated as indicated. Cells were lysed on day 0 through day 5 after differentiation and XBP1 transcription levels were measured using the real-time PCR. Knockdown efficiency is expressed relative to expression from cells transfected with control siRNA. [file 1051074.f1.docx]
